# Transient Activation of Developmental Cytoskeletal Remodeling Programs After Traumatic Brain Injury: A Multiple-Reactivation Model of Neurodegeneration

**DOI:** 10.7759/cureus.115789

**Published:** 2026-09-04

**Authors:** Steven Lehrer

**Affiliations:** 1 Radiation Oncology, Icahn School of Medicine at Mount Sinai, New York, USA

**Keywords:** arp2/3 complex, cytoskeletal remodeling, embryonic reactivation, neurodegeneration, traumatic brain injury

## Abstract

Background

Traumatic brain injury (TBI) may transiently reactivate developmental cytoskeletal programs required for neural remodeling and repair. We hypothesized that, unlike the persistent embryonic pathway reactivation proposed in amyotrophic lateral sclerosis (ALS) and Alzheimer’s disease (AD), TBI produces transient reactivation followed by suppression during recovery.

Methods

Human transcriptomic datasets were analyzed to characterize Arp2/3-mediated cytoskeletal remodeling after TBI. Single-nucleus RNA sequencing was used to assess cell-type-specific expression, and longitudinal peripheral blood transcriptomic data were analyzed at day 1 (D1), day 7 (D7), and 6 months (M6). Arp2/3 pathway scores were evaluated using differential-expression, mixed-effects, and within-subject analyses.

Results

Acute TBI showed directionally increased neuronal Arp2/3 expression. Longitudinal analysis demonstrated strong temporal variation in Arp2/3 activity (p = 2.77×10⁻⁷ in complete cases). Relative to D1, pathway activity declined at D7 (estimate -0.753, p = 0.0001) and M6 (-1.009, p < 0.0001). All 12 subjects with complete longitudinal measurements had lower M6 than D1 scores.

Conclusions

Acute human TBI brain tissue showed activation of Arp2/3-mediated cytoskeletal remodeling, while an independent longitudinal peripheral-blood cohort showed rapid attenuation and longer-term suppression of Arp2/3 pathway activity after injury. Together, these findings are consistent with, but do not establish, a transient reactivation-resolution model.

## Introduction

Traumatic brain injury (TBI) is a dynamic biological process whose consequences may evolve for months or years after the initiating mechanical insult. Injuries ranging from concussion and repetitive subconcussive impacts to severe trauma can initiate persistent structural, vascular, inflammatory, and synaptic abnormalities. Recurrent exposure is particularly relevant because cumulative mechanical injury is associated with long-term neurological and neuropathological change [1,2].

We have proposed that Alzheimer's disease (AD) and amyotrophic lateral sclerosis (ALS) involve aberrant reactivation of developmental programs that are normally downregulated after maturation [3-5]. During nervous-system development, Arp2/3-mediated branched actin polymerization, non-muscle actomyosin contractility, and microtubule remodeling generate forces required for neuronal polarization, growth-cone migration, neurite extension, and circuit formation [6]. These processes become relatively quiescent as mature neurons acquire stable architectures optimized for synaptic transmission and long-distance axonal transport.

In ALS, postmortem transcriptomic analysis has shown coordinated increases in Arp2/3 pathway expression, MYH10, and SOX9/MEGF10 developmental-glial signaling, consistent with persistent re-engagement of developmental cytoskeletal machinery. A related model has been proposed for AD, in which inappropriate reactivation of developmental motility programs may destabilize mature cytoskeletal and synaptic architecture [3,4].

TBI may provide a contrasting model. Rather than one sustained reactivation, an isolated injury could transiently engage developmental repair machinery and then suppress it as tissue remodeling resolves. The same processes used to construct the nervous system-actin polymerization, cellular migration, membrane remodeling, axonal extension, and glial responses-are also required after tissue injury [6,7]. Transient engagement may therefore be adaptive, whereas persistent or repeatedly reinitiated engagement could become maladaptive.

This distinction suggests the hypothesis of a multiple-reactivation model for repetitive TBI. Each mechanical insult could initiate a relatively small reactivation-resolution cycle. If injuries recur before recovery is complete, successive cycles could accumulate cytoskeletal, inflammatory, vascular, and synaptic consequences [8,9]. This framework does not require any single injury to reproduce AD or ALS; instead, recurrent failure to restore a stable adult state could provide a mechanistic bridge between repetitive injury and later neurodegeneration.

Longitudinal peripheral-blood transcriptomics offers an indirect means of examining whether this remodeling program changes systematically after injury. Blood cannot be assumed to reproduce central nervous system (CNS) expression, but it can capture coordinated systemic responses to TBI and recovery, and provide repeated measurements that are rarely available from human brain tissue [10]. We therefore addressed two complementary but distinct objectives. First, we analyzed acute human brain-tissue transcriptomic data to determine whether TBI is associated with increased expression of a prespecified Arp2/3-related pathway within the CNS. Second, we analyzed longitudinal peripheral-blood RNA-sequencing data collected at day 1 (D1), day 7 (D7), and 6 months (M6) after TBI to characterize the temporal trajectory of this pathway during recovery, including within-subject analyses to distinguish temporal changes from differences among independently sampled groups. The peripheral-blood analysis was considered complementary evidence of temporal regulation and was not interpreted as a direct measurement of the temporal course within brain tissue. Finally, we considered whether these findings are consistent with the hypothesis that repeated injury-associated reactivation of normally transient developmental cytoskeletal programs could contribute to cumulative neurodegenerative vulnerability. This multiple-reactivation model is proposed as a conceptual hypothesis and is not directly tested by the present datasets.

## Materials and methods

Study design

We analyzed publicly available human transcriptomic datasets to determine whether TBI is associated with temporal activation and subsequent suppression of developmental cytoskeletal remodeling pathways. The primary pathway of interest was the Arp2/3 branched-actin nucleation complex, represented by ACTR2, ACTR3, ARPC1A, ARPC1B, ARPC2, ARPC3, ARPC4, ARPC5, and ARPC5L. MYH10 and the developmental glial genes SOX9 and MEGF10 were examined as related candidate genes. This gene set was selected a priori based on its relationship to the embryonic cytoskeletal remodeling program investigated previously in ALS.

Study participants

Acute human brain transcriptomic data were obtained from GEO series GSE209552 [11]. Bulk RNA sequencing consisted of four TBI and three control brain samples. Raw gene counts were analyzed with DESeq2. Genes with fewer than 10 total reads across samples were excluded. Differential expression was estimated using a negative-binomial model with condition (TBI versus control) as the predictor. Log2 fold changes, Wald statistics, two-sided p-values, and Benjamini-Hochberg false-discovery rate (FDR)-adjusted p-values were calculated.

Single-nucleus RNA-sequencing data from the same study were additionally analyzed to determine the cellular distribution of the cytoskeletal response. Nuclei were grouped into neurons, astrocytes, microglia, oligodendrocytes, oligodendrocyte precursor cells, and endothelial cells. Gene counts were aggregated to donor-by-cell-type pseudobulk profiles to avoid treating individual nuclei as statistically independent observations. Differential expression between TBI and control donors was then evaluated within each cell type using DESeq2.

For the neuronal Arp2/3 analysis, pathway-level evidence was assessed from the constituent Arp2/3 genes. Directional consistency and the distribution of gene-level effect sizes were examined in addition to individual differential-expression statistics.

To investigate temporal behavior of the Arp2/3 program after injury, we analyzed the longitudinal human peripheral-blood RNA-sequencing dataset reported by Sadek et al. [10], publicly available through Zenodo record 18008608. The repository contains the gene-level peripheral blood mononuclear cell (PBMC) RNA-sequencing count matrix (counts_IBIS_PBMC.csv) and associated sample-level metadata (Meta_IBIS_PBMC.xlsx). The source count matrix contained 89 RNA-sequencing samples. Samples were matched to metadata using the provided sample identifier (EchantillonIdCRB); 86 of 89 samples could be matched and were retained for the primary longitudinal analysis, yielding 20 D1, 22 D7, 22 M6, and 22 healthy-control samples. The three unmatched samples, comprising one D7 and two healthy-control samples, were excluded because corresponding sample-level metadata were unavailable. Age and sex were incorporated as covariates where applicable. This metadata-matching step was distinct from the subsequent within-subject complete-case analysis. For that analysis, longitudinal subject identities were reconstructed separately from stable clinical characteristics across visits. Sixteen candidate subjects had apparent D1, D7, and M6 trajectories after duplicate removal, but only 12 subjects had unambiguous pathway-score measurements at all three time points and were therefore included in the final complete-case analysis, contributing 36 observations.

Samples were collected at D1, D7, and M6 after TBI, together with healthy control samples. Gene-expression counts and clinical metadata were matched by sample identifier. Age and sex were incorporated as covariates where applicable. Because peripheral blood is a systemic rather than CNS compartment, this analysis was prespecified as complementary evidence for temporal regulation and was not interpreted as a direct measurement of the brain response.

For each sample, an Arp2/3 pathway score was generated from the available genes in the prespecified nine-gene set. Normalized expression values were standardized separately for each gene across samples (z-scores), and the available standardized values were averaged within each sample. Higher scores therefore represented greater coordinated Arp2/3 pathway expression in standardized units.

Overall temporal differences were evaluated by regression models comparing D1, D7, M6, and healthy controls, with age and sex included as covariates. Estimated marginal means were used to calculate prespecified pairwise contrasts. Multiple-comparison correction was performed using the Benjamini-Hochberg procedure [12].

Because repeated measurements were available for a subset of participants, an additional paired analysis was performed to distinguish temporal changes from differences in the composition of independently sampled groups. Subjects were reconstructed by matching stable clinical characteristics across visits. Ambiguous matches were excluded. Twelve subjects had unambiguous measurements at all three post-injury intervals (D1, D7, and M6).

Subjects were reconstructed by matching stable clinical characteristics across visits. Ambiguous matches were excluded, and exact duplicate sample-visit rows were removed before trajectory reconstruction. Sixteen candidate subjects had apparent measurements at D1, D7, and M6; after requiring a nonmissing Arp2/3 pathway score at each of the three visits, 12 subjects remained for the complete-case repeated-measures analysis.

Arp2/3 pathway scores were analyzed using a linear mixed-effects model: $\text{Arp2/3 score}_{ij} = \beta_0 + \beta_1(\mathrm{time}_{ij}) + u_i + \epsilon_{ij}$, where i indexes participants, j indexes repeated post-injury measurements within participants, ui represents the participant-specific random intercept, and ϵij represents the residual error for measurement in participant. A likelihood-ratio test comparing models with and without the time term was used to assess the overall temporal effect. Estimated marginal means were used for D7 versus D1, M6 versus D1, and M6 versus D7 contrasts. As a distribution-free sensitivity analysis, paired Wilcoxon signed-rank tests were also performed.

A prespecified temporal trend analysis coded D1, D7, and M6 in chronological order and tested whether Arp2/3 pathway activity progressively changed with increasing post-injury interval.

Ethical considerations

This study involved secondary analysis of publicly available, de-identified human transcriptomic datasets and did not involve prospective recruitment, intervention, or direct interaction with human participants by the author. The analyses therefore did not constitute human-subjects research requiring new institutional review board approval at the author's institution. The original studies were conducted under their respective institutional ethics approvals and informed-consent procedures, as described in the source publications.

Statistical analysis

Analyses were performed in R 4.5 (R Foundation for Statistical Computing, Vienna, Austria) using data.table, DESeq2, lme4, lmerTest, and emmeans. All tests were two-sided unless a directional hypothesis was explicitly specified. A p-value < 0.05 was considered statistically significant, and Benjamini-Hochberg FDR correction was applied where multiple genes or contrasts were tested. For prespecified pairwise contrasts, Benjamini-Hochberg adjustment was applied to p-values to control the FDR, whereas Bonferroni-adjusted confidence intervals were reported to provide conservative simultaneous interval coverage across contrasts.

## Results

Bulk RNA sequencing of acute human TBI brain tissue demonstrated coordinated upregulation of multiple components of the Arp2/3 complex. Among the nine prespecified Arp2/3 genes, eight showed positive log2 fold changes in TBI compared with control tissue. ARPC1B showed the largest increase (log2FC = 3.42, fold change = 10.68, p = 0.00042, FDR = 0.021), followed by ACTR3 (log2FC = 1.41, fold change = 2.65, p = 0.00089, FDR = 0.035) and ARPC5 (log2FC = 1.40, fold change = 2.63, p = 0.00092, FDR = 0.036). ARPC2, ARPC3, and ARPC4 were also increased nominally, although they did not remain significant after FDR correction. In contrast, MYH10, SOX9, and MEGF10 were not significantly altered.

A composite Arp2/3 score was higher in acute TBI than in control brain tissue (mean standardized score, 0.556 versus -0.742; difference = 1.298). An exact permutation test across all 35 possible assignments of the seven samples yielded p = 0.0286, supporting coordinated acute activation of the pathway despite the small number of brain specimens.

Table 1 shows acute bulk-brain and single-nucleus Arp2/3 results, including log2 fold changes, p-values, and FDR-adjusted p-values.

**Table 1:** Acute brain bulk and single-nucleus RNA-sequencing data obtained from GSE209552. The longitudinal peripheral-blood dataset contained samples collected at D1, D7, and M6 after TBI and healthy control samples. The prespecified Arp2/3 pathway comprised ACTR2, ACTR3, ARPC1A, ARPC1B, ARPC2, ARPC3, ARPC4, ARPC5, and ARPC5L. Single-nucleus counts were aggregated at the donor-by-cell-type level before differential-expression analysis. For the within-subject analysis, 12 participants with unambiguous measurements at all three post-injury intervals were included. D1, day 1; D7, day 7; M6, month 6; PBMC, peripheral blood mononuclear cell; TBI, traumatic brain injury

Analysis	Biospecimen	Study design	TBI subjects/samples	Controls	Time after TBI	Primary analysis
Acute brain bulk RNA-seq	Human brain tissue	Cross-sectional	4 samples	3 samples	Acute	DESeq2 differential expression
Acute brain single-nucleus RNA-seq	Human brain nuclei	Cross-sectional, donor-level pseudobulk	12 donors	3 donors	Acute	Cell-type-specific DESeq2 pseudobulk
Longitudinal peripheral blood RNA-seq	PBMCs	Longitudinal	D1: 20; D7: 22; M6: 22	22 healthy controls	Day 1, day 7, 6 months	Arp2/3 pathway score; regression adjusted for age and sex
Complete-case longitudinal analysis	PBMCs	Within-subject repeated measures	12 subjects with all 3 visits	-	Day 1, day 7, 6 months	Linear mixed-effects model and paired Wilcoxon tests

Single-nucleus RNA sequencing further demonstrated that Arp2/3 activation was not uniform across brain cell populations. Donor-level pseudobulk analysis included 12 TBI donors and three control donors. Arp2/3 genes were predominantly increased in neurons, astrocytes, and oligodendrocytes. Six of seven detectable Arp2/3 genes were increased in neurons (mean log2FC = 0.647), all six detectable genes were increased in astrocytes (mean log2FC = 0.733), and all six detectable genes were increased in oligodendrocytes (mean log2FC = 0.556). In contrast, microglia showed no coordinated increase (mean log2FC = -0.019).

At the pathway level, donor-based Arp2/3 scores were higher in TBI neurons than in controls (difference = 0.633, p = 0.032), although this association did not remain significant after correction across cell types (FDR = 0.195). Astrocytes showed a similar but weaker increase (difference = 0.440, p = 0.094). Among selected individual genes, astrocytic ARPC5 was increased (log2FC = 1.00, p = 0.0195), whereas MEGF10 was decreased in astrocytes (log2FC = -1.23, p = 0.0278) and oligodendrocytes (log2FC = -0.96, p = 0.0223); these individual associations did not survive FDR correction.

Arp2/3 activation declines with increasing post-injury interval

The longitudinal transcriptomic dataset showed a marked temporal shift in Arp2/3 pathway activity. Relative to healthy controls, eight of nine Arp2/3 genes were increased at D1, with a mean log2FC of 0.283 and four genes significant after FDR correction. By D7, the mean log2FC had fallen to 0.073, with seven of nine genes remaining directionally increased but only two reaching FDR significance. At M6, the pattern reversed: eight of nine genes were decreased relative to healthy controls, with a mean log2FC of -0.215 and five genes significant after FDR correction.

Analysis of the composite pathway score confirmed a strong effect of time (likelihood-ratio F = 28.05, p = 1.70 × 10⁻¹² in the age- and sex-adjusted analysis). Compared with healthy controls, the adjusted Arp2/3 score was increased at D1 (estimate = 0.578, p < 0.0001), was indistinguishable from controls at D7 (estimate = -0.043, p = 0.716), and was decreased at M6 (estimate = -0.527, p < 0.0001). Pathway activity therefore declined sharply from D1 to D7 (difference = -0.621, p < 0.0001) and from D1 to M6 (difference = -1.105, p < 0.0001). A temporal trend analysis across the three post-injury intervals likewise demonstrated progressive decline in pathway activity (β = -0.548 per interval, p = 1.2 × 10⁻¹¹).

Table 2 shows longitudinal Arp2/3 pathway scores and prespecified contrasts for healthy, D1, D7, and M6.

**Table 2:** Arp2/3 pathway scores calculated from the prespecified nine-gene set ACTR2, ACTR3, ARPC1A, ARPC1B, ARPC2, ARPC3, ARPC4, ARPC5, and ARPC5L. Estimates represent adjusted differences in standardized pathway scores from the linear model including time, age, and sex. Confidence intervals were Bonferroni-adjusted for six contrasts, and p-values were corrected using the Benjamini-Hochberg procedure. D1, day 1; D7, day 7; M6, month 6; SE, standard error

Comparison	Estimate	SE	95% CI	t ratio	Adjusted p
D1 - Healthy	0.578	0.12	0.252 to 0.904	4.797	<0.0001
D7 - Healthy	-0.043	0.117	-0.361 to 0.275	-0.365	0.7158
M6 - Healthy	-0.527	0.117	-0.844 to -0.209	-4.486	<0.0001
D7 - D1	-0.621	0.12	-0.946 to -0.295	-5.160	<0.0001
M6 - D1	-1.105	0.121	-1.431 to -0.778	-9.163	<0.0001
M6 - D7	-0.484	0.117	-0.801 to -0.166	-4.121	0.0001

Table 3 shows complete-case mixed-effects analysis of the 12 subjects with measurements at D1, D7, and M6.

**Table 3:** Complete-case mixed-effects analysis of the 12 subjects with measurements at D1, D7, and M6. *Mean differences are estimated marginal mean differences from the complete-case linear mixed-effects model, with the earlier time point serving as the reference. †Benjamini-Hochberg-adjusted p-values from estimated marginal contrasts; confidence intervals were Bonferroni-adjusted. D1, day 1; D7, day 7; M6, month 6

Comparison	Mean difference*	Subjects with lower later score	Adjusted p†	Paired Wilcoxon p
D7 - D1	-0.753	10/12 (83.3%)	0.0001	0.0027
M6 - D1	-1.009	12/12 (100%)	<0.0001	0.0013
M6 - D7	-0.256	7/12 (58.3%)	0.1203	0.1277

The longitudinal pattern was reproduced when analysis was restricted to the 12 subjects with complete D1, D7, and M6 measurements. Inclusion of time significantly improved the mixed-effects model compared with the subject-intercept-only model (likelihood-ratio χ² = 30.20, df = 2, p = 2.77 × 10⁻⁷). Arp2/3 activity declined from D1 to D7 (estimated difference = -0.753, FDR-adjusted p = 0.0001) and from D1 to M6 (difference = -1.009, p < 0.0001). The additional decline between D7 and M6 was smaller and was not significant after correction (difference = -0.256, p = 0.120).

Figure 1 shows the adjusted Arp2/3 pathway trajectory across healthy, D1, D7, and M6 with 95% confidence intervals.

**Figure 1:**
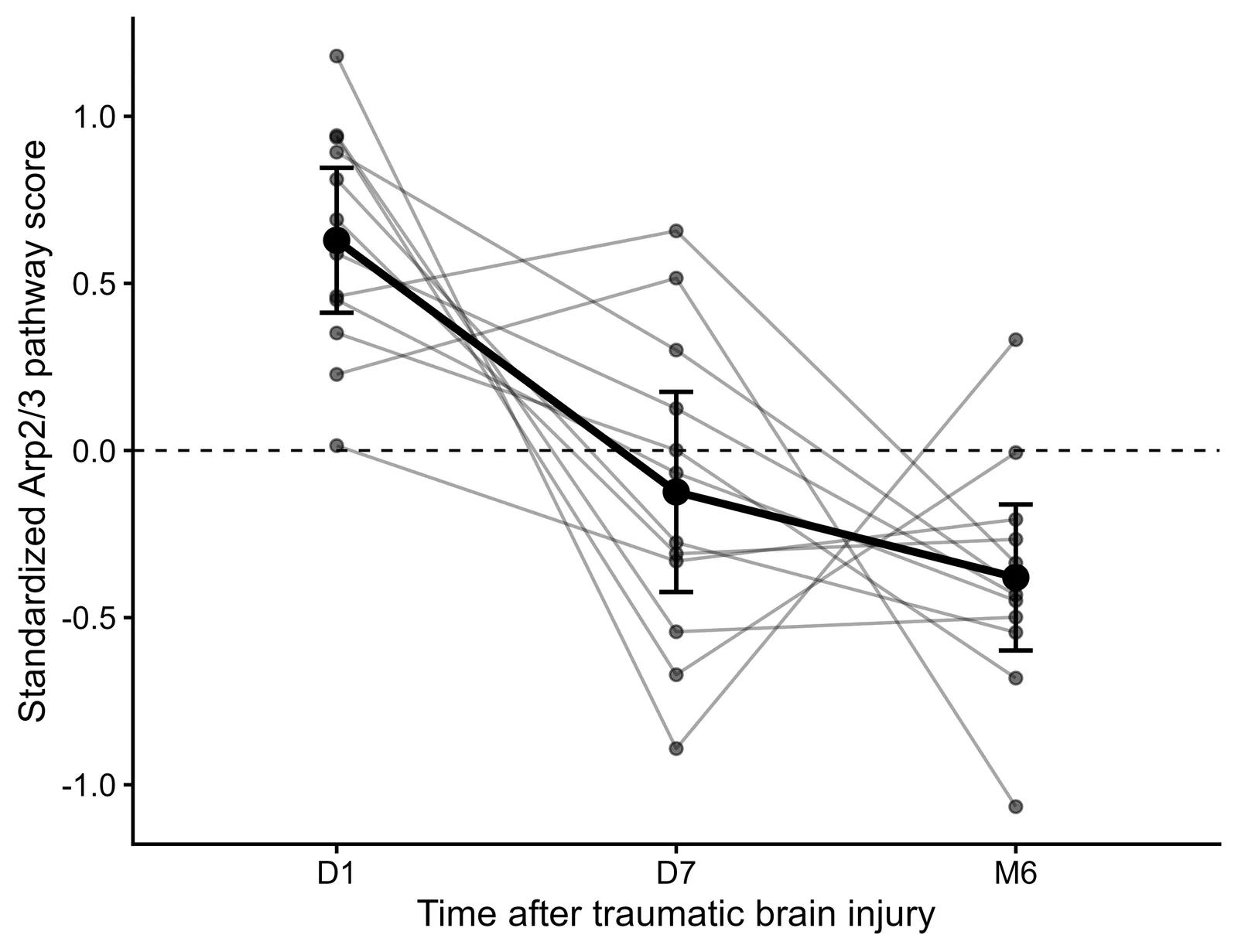
Longitudinal change in Arp2/3 cytoskeletal pathway activity after traumatic brain injury. Standardized Arp2/3 pathway scores are shown for the 12 subjects with measurements available at day 1 (D1), day 7 (D7), and 6 months (M6) after traumatic brain injury. Thin lines connect repeated measurements from individual subjects; the superimposed mean trajectory is shown with 95% confidence intervals. Arp2/3 pathway activity declined significantly over time. In the complete-case mixed-effects analysis, the estimated differences were -0.753 for D7 versus D1 (adjusted p = 0.0001) and -1.009 for M6 versus D1 (adjusted p < 0.0001). All 12 subjects had lower Arp2/3 pathway scores at M6 than at D1. The paired Wilcoxon signed-rank test for M6 versus D1 yielded p = 0.0013. The dashed horizontal line denotes a standardized pathway score of zero.

The direction of change was highly consistent among individual subjects. Specifically, 10 of 12 subjects had lower Arp2/3 scores at D7 than at D1, and all 12 subjects had lower scores at M6 than at D1. Paired nonparametric analysis confirmed lower scores at D7 versus D1 (one-sided Wilcoxon p = 0.00268) and M6 versus D1 (p = 0.00126), whereas M6 and D7 did not differ significantly (p = 0.128).

Figure 2 shows within-subject Arp2/3 pathway trajectories for the 12 participants with complete D1, D7, and M6 measurements.

**Figure 2:**
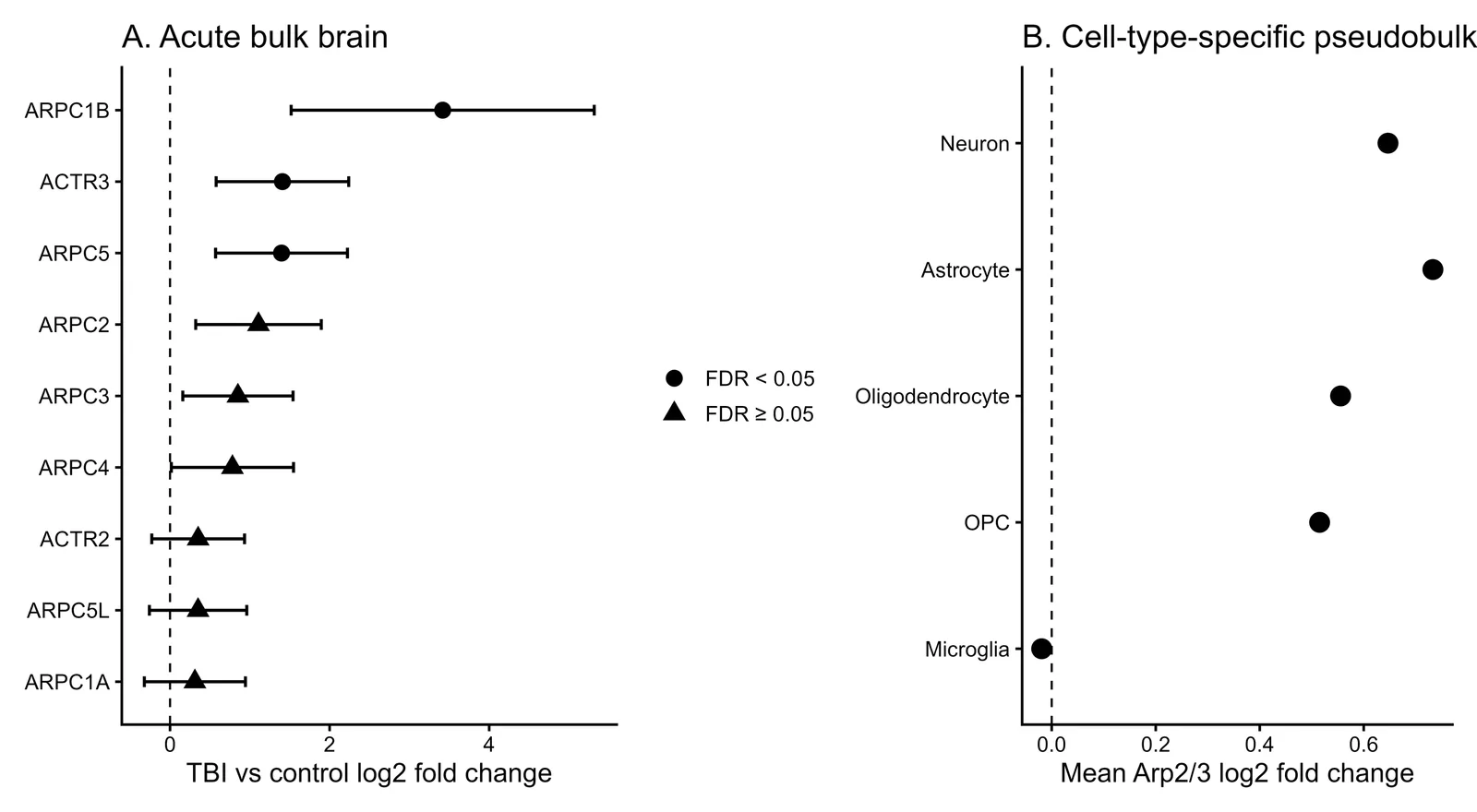
Acute activation of the Arp2/3 cytoskeletal remodeling pathway in human traumatic brain injury. (A) Differential expression of the nine prespecified Arp2/3 complex genes in acute human TBI brain tissue compared with controls. Points indicate DESeq2 log2 fold changes and error bars indicate approximate 95% confidence intervals. ARPC1B, ACTR3, and ARPC5 remained significant after Benjamini-Hochberg false-discovery-rate correction. The dashed line indicates no differential expression. (B) Cell-type-specific Arp2/3 effects from donor-level single-nucleus pseudobulk analyses. Mean Arp2/3 log2 fold changes were positive in astrocytes, neurons, oligodendrocytes, and oligodendrocyte precursor cells, whereas microglia showed no coordinated increase. These findings indicate that acute Arp2/3 activation is preferentially localized to selected neural and glial cell populations rather than representing a uniform brain-wide response. TBI, traumatic brain injury; FDR, false-discovery rate

Sensitivity analysis excluding ARPC1B. Because ARPC1B showed an unusually large individual-gene effect estimate in the acute brain analysis, we repeated the prespecified Arp2/3 pathway analysis after excluding ARPC1B. The resulting eight-gene score (ACTR2, ACTR3, ARPC1A, ARPC2, ARPC3, ARPC4, ARPC5, and ARPC5L) remained higher in acute TBI tissue than in controls (mean standardized score 0.555 vs. -0.739; difference = 1.294). The difference remained significant by exact permutation testing (two-sided p = 0.0286). Results were unchanged when the pathway was summarized using mean variance-stabilized expression rather than gene-wise standardized expression (difference = 0.763; exact two-sided p = 0.0286). Thus, the acute Arp2/3 pathway signal was not attributable to the unusually large ARPC1B effect estimate.

Taken together, the independent brain-tissue and longitudinal analyses provide convergent evidence consistent with a temporal pattern of Arp2/3 regulation after TBI. Arp2/3-mediated cytoskeletal remodeling was increased in acute brain tissue, while the longitudinal blood dataset showed marked attenuation after D1 and suppression relative to the acute state by M6. The consistent D1-to-M6 decrease within individuals argues against the longitudinal pattern being explained solely by differences in group composition. Because the analyses used different tissues and cohorts, however, they should not be interpreted as a single continuous brain time course.

## Discussion

These findings indicate that TBI is associated with temporally dynamic regulation of Arp2/3-mediated cytoskeletal remodeling. Acute human brain tissue showed coordinated pathway activation, whereas longitudinal peripheral-blood analysis showed rapid attenuation by D7 and lower activity by M6 [10]. Most notably, all 12 individuals with complete longitudinal measurements had lower M6 than D1 scores. The combined evidence is therefore consistent with transient activation followed by resolution or suppression rather than persistent pathway activation.

Although ARPC1B exhibited an unusually large individual effect estimate, exclusion of ARPC1B left the acute TBI-control Arp2/3 pathway difference essentially unchanged, arguing against a single low-expression outlier driving the pathway-level finding.

The acute brain data provide anatomical support for this interpretation [11]. Eight of nine prespecified Arp2/3 genes were directionally increased in bulk TBI tissue, with ARPC1B, ACTR3, and ARPC5 surviving FDR correction. The very large ARPC1B estimate should be interpreted cautiously because estimates from a seven-sample bulk comparison can be unstable, particularly for genes with limited expression. Single-nucleus pseudobulk analysis localized the broader signal mainly to neurons, astrocytes, and oligodendrocytes rather than showing uniform activation across cell populations [11]. Cell-type findings that did not survive FDR correction remain exploratory.

Arp2/3-mediated branched actin polymerization is central to neuronal polarization, growth-cone motility, neurite extension, membrane remodeling, and cellular migration during development [6,7]. Re-engagement after tissue damage is therefore biologically plausible because many operations required to construct the nervous system are also required to remodel injured adult tissue [6,7,9]. The important distinction may be whether this machinery is transiently deployed and successfully silenced or remains persistently dysregulated.

This distinction separates the TBI pattern from our previous observations in ALS, where Arp2/3 activity was increased together with MYH10 and SOX9/MEGF10 signaling, and from the related developmental-reactivation model proposed for AD [3,4]. TBI did not reproduce that broader molecular pattern: MYH10, SOX9, and MEGF10 were not significantly increased in acute bulk brain, and MEGF10 was nominally decreased in some cell types. Thus, physiological repair may preferentially and temporarily recruit branched-actin machinery, whereas progressive neurodegeneration may involve broader or more persistent dysregulation of developmental programs [1-4].

The results therefore refine the reactivation hypothesis: developmental pathway engagement need not itself be pathological. A single injury may generate an adaptive reactivation-resolution cycle, whereas repetitive injury could generate multiple cycles [8,9]. When injuries occur before recovery is complete, repeated engagement could progressively disturb cytoskeletal organization, axonal integrity, glial function, and synaptic stability [1,2,8]. The critical variables may be duration, magnitude, cellular localization, and the capacity to terminate the response rather than reactivation alone.

The longitudinal trajectory supports this model but also raises an unresolved question. Arp2/3 activity was highest at D1, near the healthy-control level at D7, and below the control level at M6 [10]. This apparent overshoot could represent compensatory downregulation after acute activation, but it could also reflect changing leukocyte composition, systemic recovery from critical illness, treatment, infection, or other non-neural processes [10,13]. The present data cannot distinguish these possibilities.

The within-subject analysis strengthens the evidence for a genuine temporal shift in the longitudinal cohort. Time significantly improved the complete-case model; 10 of 12 subjects declined from D1 to D7, and all 12 declined from D1 to M6. The nonsignificant M6-versus-D7 contrast after correction suggests that much of the decline occurred early. Nevertheless, reconstructed subject identities and the peripheral source of the RNA require these results to be treated as supportive rather than definitive evidence of CNS pathway resolution [10].

The model has testable implications. Prospective studies should sample the same individuals after injury at hours, days, weeks, and months and, where possible, after subsequent injuries [8,10]. Demonstration that recurrent injuries repeatedly induce and then suppress the same developmental cytoskeletal program would directly test the multiple-reactivation hypothesis. Parallel measurements in blood, cerebrospinal fluid, and brain-derived extracellular vesicles could determine whether peripheral trajectories track CNS biology.

Therapeutically, the model cautions against assuming that all early remodeling responses are harmful. If acute Arp2/3 engagement contributes to repair [6,7], indiscriminate early suppression could theoretically interfere with recovery, whereas persistent or repeatedly reinitiated activation might represent a different therapeutic target. This possibility remains speculative and requires functional and protein-level validation [14,15]. More broadly, repeated failure to restore mature cytoskeletal homeostasis could interact with tau, amyloid, neuroinflammatory, and neurovascular abnormalities that link TBI with later neurodegeneration [1,2].

Limitations

Several limitations require emphasis. First, the acute brain dataset was small, particularly the bulk RNA-sequencing comparison of four TBI and three control samples. Individual-gene estimates are consequently vulnerable to biological heterogeneity and outliers, despite the coordinated direction of the Arp2/3 findings and exact permutation analysis. The single-nucleus analysis contained more TBI donors but only three control donors, similarly limiting statistical power and precision. Several cell-type-specific findings did not remain significant after FDR correction and should therefore be regarded as exploratory.

Second, the acute brain and longitudinal datasets represent different tissues and experimental settings. The acute findings were obtained from human brain tissue, whereas the longitudinal trajectory was measured in peripheral blood. The D1-to-M6 trajectory therefore cannot establish that Arp2/3 expression within the brain follows the same time course. Peripheral expression may reflect systemic responses to injury rather than neural cytoskeletal remodeling. The convergence of the datasets supports the hypothesis, but they should not be treated as a single continuous brain time course.

Third, the longitudinal cohort includes clinical features associated with critical illness, including mechanical ventilation, pneumonia, and herpesvirus reactivation. Changes in Arp2/3 expression could therefore be influenced by systemic inflammation, infection, treatment, changing leukocyte composition, or recovery from critical illness rather than TBI alone [13]. This is an especially important limitation and precludes attributing the longitudinal blood trajectory specifically to neural injury without additional validation.

Fourth, the longitudinal subject identifiers were reconstructed from combinations of stable clinical characteristics rather than provided directly as definitive participant identifiers. Although restricting the analysis to unambiguous complete trajectories substantially strengthens the design, reconstructed matching introduces the possibility of subject misclassification. The paired analysis should therefore be described as a sensitivity analysis unless participant identity can be independently confirmed from the source study.

Fifth, pathway scores are transcriptomic surrogates. Increased expression of Arp2/3 genes does not necessarily establish increased Arp2/3 protein abundance, complex assembly, actin nucleation, or cytoskeletal force generation [14,15]. Protein-level, spatial, and functional studies will be required to determine whether the transcriptional changes correspond to activation of the molecular machinery itself.

Finally, the designation of these changes as embryonic pathway reactivation is a mechanistic interpretation rather than direct proof of developmental-state reversion. The analyses demonstrate coordinated temporal regulation of genes with established developmental cytoskeletal functions, but not epigenetic derepression of embryonically silenced loci or recapitulation of an embryonic transcriptional state. Injury severity could not be incorporated consistently across the analyzed datasets, preventing assessment of a dose-response relationship between injury severity and pathway activation. Direct comparison with fetal human neural transcriptomes, chromatin-accessibility or histone-modification data, severity-stratified cohorts, and prospective longitudinal brain or biofluid studies would provide stronger tests of the hypothesis.

## Conclusions

In conclusion, acute human TBI brain tissue showed activation of Arp2/3-mediated cytoskeletal remodeling, while an independent longitudinal peripheral-blood cohort showed rapid attenuation and longer-term suppression of Arp2/3 pathway activity after injury. Together, these findings are consistent with, but do not establish, a transient reactivation-resolution model.
